# Exploring the Influencing Paths of Villagers’ Participation in the Creation of Micro-Landscapes: An Integrative Model of Theory of Planned Behavior and Norm Activation Theory

**DOI:** 10.3389/fpsyg.2022.862109

**Published:** 2022-06-29

**Authors:** Huishan Cheng, Quanquan Rui, Kunyong Yu, Xiaohe Li, Jian Liu

**Affiliations:** ^1^College of Landscape Architecture, Fujian Agriculture and Forestry University, Fuzhou, China; ^2^Haixia (Fujian) Transportation Engineering Design Co., Ltd., Fuzhou, China; ^3^College of Forestry, Fujian Agriculture and Forestry University, Fuzhou, China

**Keywords:** rural micro-landscape construction, influence factors, planned behavior theory, normative activation theory, AMOS

## Abstract

Villager participation has become a key breakthrough in rural landscape governance. Using the theory of planned behavior and the norm activation theory as frameworks, this study adopts the structural equation model to explore the influencing mechanism of villager participation in rural micro-landscapes based on data gathered from 414 villagers in a rural micro-landscape construction survey in Jinjiang, China. The results indicate that (1) integrated planned behavior theory and norm activation theory can better explain the influencing mechanism of villagers’ participation in rural micro-landscape construction; (2) perception, norm, attitude, and control dimensions significantly influence villagers’ participation behavior intention. The attitude dimension had the greatest influence, followed by the normative and control dimensions, while the perception dimension had the least influence on the procedure; and (3) according to the mediation results, natural environment perception, social environment perception, personal norm, social norm, participation attitude, result awareness, and self-efficacy all exert indirect effects on participation behavior based on villagers’ participation behavioral intention. The largest median effect value was result awareness, followed by personal norm, participation attitude, natural environment awareness, self-efficacy, and social norm. This study expands the theoretical framework and research content of planned behavior and clarifies the mechanism of the influencing factors of villagers’ participation in rural micro-landscapes, extending the theory of planned behavior to the research field of villagers’ participation, which has a guiding role in promoting the co-construction, co-governance, and sharing of rural landscapes.

## Introduction

Villager participation is not only a major method to maintain the balance of rural interests and promote the efficacy of planned rural construction but also a means to provide essential support for rural vitalization (Buchecker et al., 2003; Qian et al., 2016; Cheng et al., 2019). The importance of villager participation in rural construction was emphasized at the 19th National Congress of the Communist Party of China and the No. 1 Central Document in 2018. However, the problems caused by low villager participation and poor participation performance are still prevalent (Kusters et al., 2018; Peifen, 2021; Yun, 2021). Therefore, enhancing villager participation and facilitating individual villager participation in rural landscape construction is an issue highlighted by both government and theoretical circles. Nevertheless, the decision-making process of villagers regarding the construction of rural landscapes is extraordinarily complicated. Although some villagers are extremely concerned about the rural landscape, they may be more concerned about other factors, such as interests and hobbies, when choosing whether to participate. The question of whether villagers choose to participate in rural landscape construction is worthy of in-depth discussion. What are the factors that affect villagers’ participation in rural landscape construction? Which factors are the most decisive? Research on these issues will contribute to finding effective ways to improve villagers’ participation behavior, meet villagers’ demands for rural landscapes, and provide decision-making references for government departments to improve the stickiness of rural communities.

The research results on public participation in rural planning mainly focus on the following four aspects: participation systems (Santé et al., 2020; Balest et al., 2021), the need for participation (Fleischman and Solorzano, 2018; Staniewska, 2018; Palmer et al., 2022), the methods and techniques of participation (Brown and Kyttä, 2014; Eiter and Vik, 2015; Palacio Buendía et al., 2021), and the restrictive factors of participation (Lyons, 2005; Ross et al., 2016). Studies performed by numerous scholars on the influencing factors of villagers’ participation behavior have primarily concentrated on exterior structures (Folz and Hazlett, 1991; Lawrence et al., 2020), social demography (Maloney and Ward, 1973; Shi, 2016), and social psychology (Greaves et al., 2013; Chen et al., 2020; Hou et al., 2021). Folz and Hazlett (1991) investigated the application of public regulation in solid waste and showed that, among many influencing factors, community regulation has a significant impact on the recycling of solid waste. By analyzing data from the Chinese General Social Survey in 2010 (CGSS2010), Shi (2016) found that individuals of different classes exhibited significantly different environmental behaviors. Zhou (2018) constructed a model of the influencing factors of villagers’ environmental behavior based on the theory of planned behavior (perception, attitude, subjective norms, and perceptual behavior control) and highlighted the local government’s deficiencies in environmental quality and improvement measures. These results provide a solid foundation for this research; however, some persistent problems remain to be addressed. Most existing studies introduce one or two variables for further analysis using the theory of planned behavior, the norm activation theory, and the value-norm-belief theory; however, considering the lack of examination of all theoretical variables under comprehensive framework construction, it is impossible to explore the factors influencing villager participation accurately and effectively.

To fill this gap in the research, this study considers the theory of planned behavior as the research framework and integrates the theory of norm activation, the theory of value-belief-norm, and the social cognition theory to explore the determinants of villagers’ participation in the creation of a rural sense of crisis. The aims of this study are two-fold: (1) to identify the factors affecting villagers’ participation in micro-landscape creation activities and (2) to determine which factors are more decisive. This study proposes an effective theoretical framework to explain the factors that affect villagers’ participation in rural micro-landscape creation. Moreover, this research proposes useful management strategies for villager participation to achieve the sustainable development of rural society. Compared to previous research in the field of public participation, this study combines the theory of planned behavior, the norm activation theory, the value-belief-norm theory, and the social cognition theory to explore whether villagers’ participation in rural landscape construction is an original approach.

Taken together, the contributions of this study are three-fold. First, we consider the theory of planned behavior as the research framework for the first time, synthesize the norm activation theory, the value-belief-norm theory, and the social cognition theory, and construct a conceptual model of villagers’ participation behavior in rural micro-landscape creation from the perspective of villager participation. The factors and mechanisms that affect villagers’ participation behavior expand the application boundaries of the theory of planned behavior. Second, 414 survey answers from villagers in Jinjiang City were used to conduct the empirical research. The use of first-hand data ensured that the research findings were reliable and reasonable. As the actual effect of villagers’ participation in micro-landscape construction is discussed, our research results have practical significance and social value. Lastly, based on the findings of the study, we make recommendations for achieving sustainable development. Thus, the findings presented in this study can help policymakers create practices that increase villager participation and achieve the sustainable development goals of rural communities. In addition, this study also provides theoretical support and empirical evidence for strengthening villagers’ participation and promoting rural revitalization, providing scientific suggestions for the development, design, construction, and innovation of rural communities.

The remainder of this article is organized as follows. See section “Literature Review” reviews the relevant literature on the theory of planned behavior, the norm activation theory, the value-belief-norm theory, and the social cognitive theory. See section “Conceptual Model and Research Hypotheses” constructs the relational model of villagers’ participation in micro-landscape construction and proposes the research hypotheses. See section “Research Area Overview and Research Design” presents the study design and outlines the data collection process. See section “Empirical Analysis” explains the results of model checking and the resulting equation model analysis. See section “Results and Analysis” summarizes the key factors affecting villagers’ participation in rural landscape construction. Then, a discussion of the research results is presented. In the final section, conclusions, recommendations, and the limitations of this study are discussed.

## Literature Review

As briefly mentioned in the Introduction, this section analyzes four theories: the theory of planned behavior, the norm activation theory, the value-belief-norm theory, and the social cognitive theory.

### Theory of Planned Behavior

The theory of planned behavior (TPB) expands on Ajzen’s theory of rational behavior (TRA) by adding the variable of perceptual behavior control (Ajzen, 1985). TPB is one of the most important theories concerning individual behavior generation in social psychology. The theory considers behavioral intention to be the direct factor that determines behavior. At the same time, behavioral attitude, subjective norms, and perceived behavior control are three variables that can affect behavioral intention. Behavioral intention is a summary of the advantages and disadvantages of an individual’s inner judgment of whether to consider performing a certain behavior. Attitude refers to the evaluation of the approval or disapproval of a behavior. Subjective norms refer to an individual’s perception of whether a particular behavior is a type of social pressure that reflects the influence of important others or groups on individual behavior decisions, such as the views of family, friends, and colleagues. Perceived behavioral control refers to the difficulty of an individual’s perception of specific behavior and reflects their views on factors that promote or hinder the execution of the behavior (Ajzen and Driver, 1991; Ajzen, 2004).

Since TPB was first proposed, it has been extensively applied to explain and predict the occurrence of various behaviors. TPB has also been widely used in human and co-participation behavior research (Chen et al., 2020; Empidi and Emang, 2021; Li et al., 2021). For example, Zeng (2017) used TPB as the research framework to analyze the feasibility and difficulties of public participation in environmental governance and constructed a participation mechanism of “empowerment-recognition-cooperation” to improve the level of public participation. Research has shown that the public’s attitude toward environmental protection positively affects their willingness to participate in environmental protection behaviors (Zheng et al., 2017).

### Norm Activation Theory and Value-Belief-Norm Theory

Before the introduction of the norm activation theory, most psychologists believed that social or material rewards were the factors that promoted individuals to engage in altruistic behavior. The norm activation theory proposed by Schwartz (1977) is considered important for the study of altruistic behavior in the field of social psychology. This theory is composed of three variables: awareness of consequences, the ascription of responsibility, and personal norms. The consciousness of the result refers to an individual’s awareness of the result of not engaging in altruistic behavior, as well as the consciousness of causing adverse consequences to others. Under normal circumstances, the stronger the individual’s perception of the results of a particular situation, the stronger the obligation to arrive, and the more likely the individual will activate individual norms to implement the corresponding altruistic behaviors. The attribution of responsibility refers to an individual’s sense of responsibility for adverse consequences. The stronger the individual’s sense of responsibility or awareness of the results, the more conducive they are to the production of personal behavior. That is, the stronger the awareness of the results, the more conducive it is for individuals to produce behaviors that conform to their personal norms. For example, when people think that protecting the environment is good for their health, some will regulate their environmental behavior and take pride in it. This is also a manifestation of social norms and self-morality. Thus, personal norms are internalized by social norms and a sense of self-moral responsibility. Violating personal norms can lead to guilt, self-denial, or a loss of self-esteem as personal norms create a sense of pride and self-esteem.

Since the introduction of norm activation theory, it has been widely used in altruistic behavior research and has been demonstrated to have significant explanatory and predictive power. However, in theory, the relationship between the main variables is constantly being adjusted. There are two relationships between the variables: the moderation model, which influences the behavior of individual norms, and the mediation model, which indicates that awareness of an outcome influences individual norms through responsibility attribution (Schwartz, 1973). Based on the norm activation theory, Onwezen et al. (2013) used an intermediary model to explore the public’s environmental behavior. Lu (2016) researched the factors influencing individual energy-saving behavior based on the extended norm activation theory. Empirical evidence shows that extended norm activation theory can be successfully applied to the study of individual energy-saving behavior. Stern et al. (1999) adopted the mediation model of norm activation theory to combine value theory with new environmental paradigm viewpoints and scales, proposing value–belief–norm theory (VBN). This theory is based on the application and development of norm activation theory in the field of public pro-environmental behavior research, which is considered to be the best theory for investigating various environmental pro-environmental behaviors. VBN theory connects the five variables of personal value orientation, NEP, result awareness, responsibility attribution, and individual norms through causality. In this causal relationship, each variable directly affects the next variable and influences subsequent variables. The value–belief–norm model includes three aspects, namely egoism, altruism, and ecological value, avoiding the unity of norm activation theory based on altruism (Onwezen et al., 2013). Ibtissem (2010) analyzed the public’s energy-saving behavior based on the value-belief-norm theory. Empirical evidence demonstrates that individual value orientation significantly affects awareness of the results. Awareness of the results influences individual norms through responsibility attribution, and individual norms directly affect individual energy-saving behaviors. Steg et al. (2005) analyzed the factors influencing energy policy to lower household carbon dioxide emissions and verified the VBN theory.

### Social Cognitive Theory

In the 1980s, the American scholar Bandura (1977) proposed the theory of social cognition, which mainly includes ternary interactive determinism, observational learning, and self-efficacy. Ternary interactive determinism refers to human behavior, environmental factors, and individual factors. Determinism considers the relationship between these three to be independent and interactive. Bandura proposed that the behavior of an individual is affected by the behavior of others. Observational learning indicates that individuals can generate new behaviors in their learning process by observing the behaviors or habits of others, as well as improving and perfecting their original behaviors in this process. Self-efficacy refers to an individual’s self-judgment of their ability to complete a certain activity at a certain level, including their beliefs, feelings, and mastery. Self-efficacy can be understood as an individual’s self-confidence and ability to complete an action. Therefore, self-efficacy can also be called self-confidence. Although there are several related results, with numerous studies being conducted on individual social learning behaviors, few studies have explored pro-environmental behaviors and influencing factors.

## Conceptual Model and Research Hypotheses

Currently, studies on personal behavior participation theory mostly involve classical theories in the fields of psychology and information systematics (Fu et al., 2021), such as TPB (Amit Kumar, 2021; Wang et al., 2021), the norm activation theory (He and Zhan, 2018; Zhao et al., 2019), VBN (Stern, 2000; Kiatkawsin and Han, 2017; Granco et al., 2019), and the social cognition theory (Dzewaltowski et al., 1990; Deng et al., 2016; Hagger et al., 2017). Scholars, such as Wynveen and Sutton (2015), believe that TPB should be able to explain individual behavior more accurately than other theories related to behavior research. However, simultaneously, we should try to combine the research results of other theories and add new variables to enhance the explanatory power of the model. Therefore, to further explore the association among personal behavior theories, this research makes a comparative study based on the influencing factors and characteristics of personal behavior, as shown in Table 1.

**TABLE 1:** Comparison of the main theories of personal behavior research.

Name of theory	Theory of planned behavior	Norm activation theory	Value- belief-norm theory	Social cognition theory
Influencing factors of personal behavior	attitude	result awareness	value orientation	observing learning
	subjective norm	attribution of liability	NEP	self-efficacy
	perceived behavior control	personal norm	result awareness	environment perception
			attribution of liability	
			personal norm	
Characteristics	begin with self-interest	Prioritize altruism	Combine altruism, egoism, and ecological value	Observe social learning behavior
				

As shown in Table 1, the norm activation theory prioritizes altruism, whereas TPB is based on personal self-interest. As a result, neither theory has been directly applied in this research. In contrast, VBN integrates three value orientations: altruism, self-interest, and ecological value. Due to the low income and education level of villagers, throughout the process of investigation, their altruistic motivations and consideration for ecological value were found to be very weak. Meanwhile, their personal behaviors were essentially out of self-interest. Therefore, the VBN cannot be fully applied to the study of villagers in Jinjiang.

Personal norm has the most direct influence on individual behavior, and the norm activation model is considered an effective model for studying public participation behavior (Zhang, 2016). In TPB, subjective norm mainly refers to the social pressure on individuals, and in the process of micro-landscape construction, the participation behavior of villagers is not only affected by social norms but also by their personal norms. Therefore, in this study, subjective norms in TPB are divided into personal and social norms; that is, the norm dimension includes both personal and social norms. The norm activation theory argues that an awareness of consequences directly affects individual norms. Schwartz (1980) studied the mutual aid behavior of college students using the norm activation model and found that the weaker the individual’s awareness of the results, the smaller the impact on behavior. In discussing the behavior of residents participating in environmental improvement, Zhou (2018) pointed out that many individuals have different attitudes toward the environment due to differences in their awareness of consequences, and incorporated the attitude of participation and awareness of results into the attitude dimension. The present study followed this method and divided the attitude dimension into participation attitude and result awareness. Ajzen (2002) further explained perceptual behavioral control and believed that perceived behavioral control could not be simply divided into two parts. He pointed out that its essence should be the individual’s confidence in completing the behavior and named it self-efficacy. Based on this, the study adopts Ajzen’s (2002) specific explanation of perceived behavioral control and uses self-efficacy as a measure of the control dimension. Its specific meaning is the size and confidence of residents, which plays a role in their participation in landscape construction. According to the relevant research by Mesch and Manor (1998), this study also believes that, in addition to social environment perception (SEP), NEP may be an important factor affecting villagers’ participation in landscape construction. It should thus also be included in the model as a perception dimension.

**FIGURE 1:**
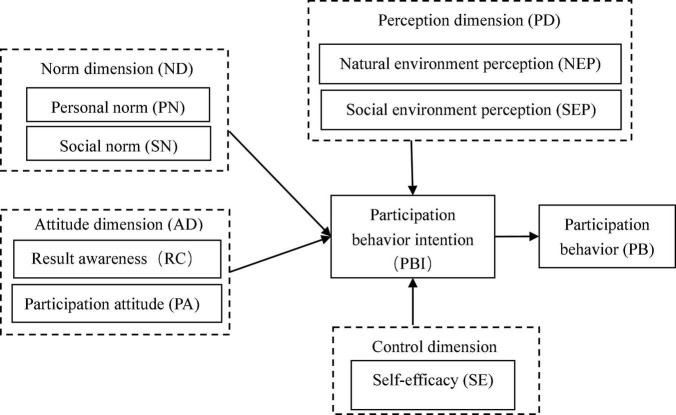
Integrated theoretical framework.

In this context, this study attempts to use TPB as the research framework while drawing on and referring to the influencing factors of individual behavior in the other three theories, retaining the three main dimensions of the norm dimension, attitude dimension, and control dimension in TPB, with the addition of the perception dimension. As a result, a research model of villagers’ participation in rural landscape construction in Jinjiang City was constructed (Figure 1). The sources of these indicators are listed in Table 2.

**TABLE 2:** Metric sources for research models.

Indicator dimension	Indicator source	Related research
Natural environment perception	Social cognition theory	Peng and Zhou, 2001; Liu, 2021
Social environment perception	Social cognition theory	Craik, 1972; Guo et al., 2015; Wang and Wu, 2015; Ruan, 2020
Personal norm	Theory of planned behavior Norm activation theory Value-belief-norm theory	Ajzen, 1985; Cialdini et al., 1990; Wang and Wu, 2015; Zhang, 2016
Social norm	Theory of planned behavior	Ajzen, 1985; Turner et al., 1991; Nolan, 2008
Participation attitude	Theory of planned behavior	Shaw et al., 2000; Daniel and Klaus, 2022
Result awareness	Value-belief-norm theory	Schwartz, 1980
Self-efficacy	Theory of planned behavior Social cognition theory	Bandura, 1977, 1982; Ajzen, 2002
Participation behavior intention	Theory of planned behavior Norm activation theory	Schwartz, 1980; Ajzen, 1985

Each dimension and indicator are discussed in the following sections.

### Perception Dimension

The research subject of this article is villagers who show an intense perception of changes in their local living conditions. Based on the formation of environmental factors, Mesch and Manor (1998) classified the perception dimension (PD) into NEP and SEP. Accordingly, personal empathy for the natural environment can trigger two forms of environmental behavior: the personal form, as reported by Lin et al. (2017); Xiu (2021), and Ying (2021), and the social form, which includes participation in social environmental protection organizations (Armitage and Conner, 2001; Berenguer, 2010). This study follows the aforementioned research, subdividing the PD of villager participation in rural micro-landscape construction into NEP and SEP. The following hypotheses are proposed:

**H1:** There was a significant positive influence between the perception dimension and participation behavior intention (PD→ PBI).
**H1(a):** There was a significant positive relationship between NEP and villager participation behavior intention (NEP→ PBI).
**H1(b):** There was a significant positive relationship between SEP and villager participation behavior intention (SEP→ PBI).

### Norm Dimension

According to planned behavior theory, subjective norms refer to social pressure on individuals (Ajzen and Driver, 1991; Berki-Kiss and Menrad, 2022; Dai et al., 2022). Norm activation theory points out that personal norm has the largest effect on personal behavior (Schwartz, 1973; Shi et al., 2017; Song et al., 2019). Based on a literature review of planned behavior theory and norm activation theory, this study divides subjective norms into personal norm (PN) and social norm (SN; Cialdini and Goldstein, 2004). Research indicates that PN would directly affect individuals’ behavioral intentions to enhance or maintain the environment (Hallin, 1995; Wynveen and Sutton, 2015). Thus, in the process of the construction of rural landscapes, the higher the self-regulation of villagers, the stronger their participation intention. In contrast to PNs, SNs refer to the behavioral norms observed by individuals or organizations (Schwartz, 1977; Terrier and Marfaing, 2015). Based on the above analytical results, the following hypotheses are proposed:

**H2:** There was a significant positive influence between the norm dimension and participation behavior intention (ND→ PBI).
**H2(a):** There was a significant positive influence between personal norm and villager participation behavior intention (PN→ PBI).
**H2(b):** There was a significant positive influence between social norm and villager participation behavior intention (SN→ PBI).

### Attitude Dimension

Planned behavior theory proposes that individual behavior is primarily affected by attitudes, subjective norms, and cognitive-behavioral control (Boldero, 1995; Kaiser and Gutscher, 2003). Norm activation theory posits that the resulting awareness directly manipulates PN (Nyborg, 2018). The value–belief–norm model argues that personal behavior is affected by PN and the result awareness (Vining and Ebreo, 1992; Bratt, 1999). Fishbein and Ajzen (1977) pointed out that participation attitude (PA) is a continuous and persistent positive (like) or negative (dislike) feeling that an individual has toward a specific thing or problem. Using the planned behavior theory and norm activation theory as research frameworks, Wang and Zhang (2017) verified that individuals’ attitude toward participation positively affected their participation behavior intention. Result awareness (RC) is an individual’s judgment of the consequences of behavior or policy after it has been implemented. Through the perception of environmental problems, individuals can establish an awareness of the results, which can promote their behaviors to participate more actively in landscape construction; in turn, this can help alleviate or reduce environmental problems. Previous studies have demonstrated that the stronger the awareness of certain behaviors, the stronger the sense of morality. In this case, individuals are more likely to engage in personal behavior (WESLEY Schultz and Zelezny, 1999). On this basis, this study adopts result awareness and PA as research variables and proposes the following hypotheses:

**H3:** There is a significant positive influence between the attitude dimension and participation behavior intention (AD→ PBI).
**H3(a):** There was a significant positive relationship between participation attitude and villager participation behavior intention (PA→ PBI).
**H3(b):** There was a significant positive relationship between participation result awareness and villager participation behavior intention (RC→ PBI).

### Control Dimension

The original control dimension in TPB is perceived behavioral control. Bandura (1977) proposed self-efficacy to specifically refine perceived behavioral control. Subsequently, Ajzen (2002) proposed that the essence of perceived behavioral control is an individual’s confidence in completing behaviors, which they denoted as self-efficacy (SE). Based on this, the present study uses SE as a measure of the control dimension, which specifically refers to the size and confidence of residents that they can play a role in participating in landscape construction. Studies have shown that SE can promote the action of recycling at an individual level by influencing environmental behavior (Bandura, 1977, 1982; Ajzen, 2002). When faced with problems, individuals with greater SE tend to demonstrate positive aspects, thus promoting the development of the event toward success (Tsiligianni et al., 2020; Yu et al., 2020). Therefore, in the construction of rural landscapes, villagers with stronger SE are often more willing to participate. Based on the above analysis, this study proposes the following hypothesis:

**H4:** There was a significant positive relationship between SE and villager participation behavior intention (SE→ PBI).

### Behavior Intention

Participation behavior intention (PBI) refers to personal intention and effort in making an attempt, which is the intrinsic driving force of personal behavior (Wang et al., 2020). TPB believes that the behavioral intention of the behavior subject is the best way to predict the behavior. Based on TPB, Xiao and Wang (2017) verified that farmers’ willingness to participate in behavior positively affects their participation behavior, and some scholars believe that willingness to participate in behavior directly determines participation behavior. Therefore, the stronger the villagers’ willingness to participate in rural landscape construction, the greater the possibility of their participation. Although planned behavior theory has been demonstrated to be effective in explaining and predicting personal behavior, there are still many behaviors that cannot be completely rendered in practical surveys (Kaiser, 2006; Bortoleto et al., 2012; Hu et al., 2018). According to the research model, villager participation behavior can be divided into two stages: motivation and implementation. Does PBI play a mediating role between motivation and behavior? Accordingly, the following hypotheses are proposed:

**H5:** There was a significant positive relationship between participation behavior intention and villager participation (PBI →PB)
**H6:** Villager PBI in rural landscape construction played a mediating role between antecedent variables and participation behavior.

## Research Area Overview and Research Design

### Research Area Overview

The city of Jinjiang is located on the southeast coast of Fujian Province and is at the heart of the Golden Triangle of southern Fujian. With China’s “new socialist countryside” construction (16th National Congress), “beautiful countryside” construction (18th National Congress) and the development strategy of “rural revitalization” (19th National Congress), China’s rural construction began from the outside in, characterized by in-depth ecological protection, cultural heritage, and industrial revitalization. As a pilot project for the creation of a beautiful countryside in China, Jinjiang continues to promote the construction of rural landscapes through micro-landscape creation. Thus far, 42 city-level Jinjiang and over 100 rural micro-landscapes have been established. Although the micro-landscape construction activity in the city of Jinjiang only started 2 years ago, it already had a significant effect on rural landscape construction in Jinjiang. Its fundamental role is to fully mobilize the enthusiasm of the local villagers, increasing the stickiness of rural communities and providing a new model for rural beautification and enhancing the heritage of rural culture. With characteristics including reproducibility, promotion, and positive effects, the city of Jinjiang in China represents a site with a high practical research significance.

### Variable Measurement

Based on both national and international research results, this study designed a variable-measurement scale (Table 3). NEP and SEP primarily used the scale of Keshuai (2008), while PN perception used the scale of Gärling et al. (2003) and SN used the scale of Cialdini and Goldstein (2004) and Bissing-Olson et al. (2016). PA used the scale of Lee et al. (2014), while result awareness conformed to the scale of Onwezen et al. (2013). Self-efficiency was measured using the scale of Chen and Hung (2010), while the scale of Matthew (2017) was adopted for participation behavior intention and participation behavior. Responses to research questions were measured using a five-point Likert scale (1 = strongly disagree to 5 = agree).

**TABLE 3:** A survey project on villagers’ participation in rural micro-landscape construction.

Variable	Items
**Natural environment perception (NEP)**	
NEP1	My overall satisfaction evaluation of the current village construction
NEP2	I hope to improve the relationship between people and the environment by participating in rural micro-landscape creation activities
NEP3	I hope that by participating in rural micro-landscape creation activities, the quality of the environment can be improved
**Social environment perception (SEP)**	
SEP1	The government will pay great attention to the interests of participants in micro-landscape activities
SEP2	The village committee advocates micro-landscape creation activities
SEP3	I hope to promote the relationship between village neighbors by participating in micro-landscape creation activities
**Personal norm (PN)**	
	In general, in the past in the process of participating in micro-landscape activities
PN1	I find it interesting
PN2	I feel happy
PN3	I feel a sense of personal achievement
**Social norm (SN)**	
SN1	Many neighbors have participated in micro-landscape creation activities
SN2	Many people think that I should participate in micro-landscape creation activities
SN3	Relatives and friends think I should participate in micro-landscape creation activities
**Participation attitude (PA)**	
PA1	Participating in rural micro-landscape creation activities is good for me
PA2	Participating in rural micro-landscape creation activities is valuable to me
PA3	It is wise for me to participate in rural micro-landscape creation activities
**Result awareness (RC)**	
RC1	I hope to improve the relationship between people and the environment by participating in rural micro-landscape creation activities
RC2	The construction of rural micro-landscape can be conducive to the harmonious coexistence of man and nature, mutual assistance and trust between people
RC3	Rural micro-landscape is conducive to the recycling and reuse of waste resources
**Self-efficacy (SE)**	
SE1	I have the skills and knowledge to complete the creation of rural micro-landscapes
SE2	I have the resources needed to complete the creation of rural micro-landscapes
SE3	If invited, I can complete micro-landscape creations
**Participation behavior intention (PBI)**	
WT1	I am willing to obtain information on rural micro-landscape construction
WT2	I am willing to participate in the related work of rural micro-landscape construction (project discussion, data collection, construction beautification, maintenance, etc.)
WT3	I am willing to participate in rural social activities
**participation behavior (PB)**	
PB1	I will know about the development status of rural micro-landscape creation from newspapers, TV or the Internet
PB2	I would love to participate in micro-landscape activities with people I know
PB3	I am happy to use my free time to participate in rural micro-landscape creation activities

### Data Collection

A field survey was conducted in the Jinjiang countryside from 25 December 2018 to 30 December 2018. A total of 450 questionnaires were issued and 414 valid questionnaires were returned, with an effective rate of 92%. Although the structural equation model is applicable to large-sample research, the survey questionnaire cannot be infinitely large. Therefore, for the sample size of the survey, this study adopted the viewpoint proposed by Thompson (Thompson, 1998); that is, the ratio of respondents to measurement items should be at least 10:1–15:1. Among the valid samples, 47.3% were men and 52.7% were women. Therefore, the proportion of males and females was roughly equivalent. Most research participants were aged 31–40 and 41–50, accounting for 30.2 and 29.9% of the cohort, respectively. The vast majority of participants had a middle school to high school education. SPSS19.0 and AMOS24.0 were used to process the data and verify the conceptual model.

## Empirical Analysis

### Common Method Deviation Control and Test

In this study, the Harman single-factor method and partial correlation coefficient method were adopted for questionnaire analysis (Eby and Dobbins, 1997; Livingstone et al., 1997; Podsakoff et al., 2003). A total of seven common factors with eigenvalues greater than one under non-rotated conditions were obtained, among which the variance contribution rate of the first common factor was 34.19% (i.e., less than 40%), indicating that the common method deviation of the questionnaire measurement scale was insignificant and appropriate for subsequent data analysis.

### Descriptive Statistical Analysis and Validity and Reliability Test

According to the standard of normal distribution proposed by Wu (2009), the absolute value of the skewness coefficient should be less than 3, and the absolute value of the kurtosis coefficient should be less than 10. The closer the coefficient values of the skewness and kurtosis coefficients are to 0, the more they conform to a normal distribution. As shown in Table 4, there was no significant difference in the standard deviation of all observed variables. A standard deviation value in the range of 0.544–0.881 can ensure high identification. The corresponding deviation coefficient was in the range of −1.294–0.297, while the kurtosis coefficient was in the range of −0.975–3.724, proving that the sample data approached the normal distribution. At present, Cronbach’s alpha coefficient is often used to test the internal consistency of variables (Liu et al., 2020). As shown in Table 4, Cronbach’s α coefficient of each latent variable was greater than 0.7, while that of the gross scale was 0.938. All composite reliability (ρ_*c*_) values were higher than 0.7, proving the reliability of internal consistency, according to Esbensen et al. (2009). The average variance extracted (AVE) was used to explain the convergent validity of the measurement index. Except for the latent variable PA (AVE = 0.464), the AVE of the remaining latent variables was above 0.5. In confirmatory factor analysis, standardized regression coefficients, also known as factor loadings, represent the influence of common factors on measured variables. Except for the PA1 standardized coefficient of 0.459, the standardized factors of all observed variables were above 0.5, and significant at the level of 0.01, demonstrating the favorable convergent validity of the scale. By comparing the AVE square root, row, and column correlation coefficients of all latent variables in Table 5, the square root of AVE was the maximum value of its row and column, indicating that the latent variables had a high distinction validity. The critical ratio (C.R.) value is equal to the ratio of the unstandardized estimate to Standard Error (S.E.), which is equivalent to the *t*-test value. If the absolute value of this value was greater than 1.96, the *P*-value reached a significance level of 0.05, denoted by the symbol “*”; if the absolute value was greater than 2.58, the *P*-value reached a significance level of 0.01, denoted by the symbol “^**^”; if the absolute value was greater than 3.25, the *P*-value reached a significance level of 0.001, denoted by the symbol “^***^”.

**TABLE 4:** Analysis of the reliability and validity of the villagers’ participation behavior model.

Latent variable	Measurement item	Standard deviation	Unstandardized estimate	S.E.	C.R.	*P*	Standardized estimate	ρ_*c*_
Natural environment perception (NEP)	NEP1	0.833	1.000				0.655	0.751
	NEP2	0.634	1.078	0.325	3.316	***	0.829	
	NEP3	0.647	0.747	0.212	3.532	***	0.631	
Social environment perception (SEP)	SEP1	0.718	1.000				0.761	0.815
	SEP2	0.680	1.156	0.235	4.916	***	0.828	
	SEP3	0.661	0.875	0.188	4.645	***	0.724	
Personal norm (PN)	PN1	0.671	1.000				0.897	0.905
	PN2	0.726	1.083	0.144	7.529	***	0.898	
	PN3	0.595	0.808	0.121	6.686	***	0.818	
Social norm (SN)	SN1	0.647	1.000				0.829	0.831
	SN2	0.790	1.308	0.274	4.769	***	0.888	
	SN3	0.821	0.969	0.238	4.073	***	0.633	
Participation attitude (PA)	PA1	0.682	1.000				0.459	0.711
	PA2	0.634	1.421	0.522	2.723	**	0.702	
	PA3	0.593	1.758	0.773	2.276	*	0.829	
Result awareness (RC)	RC1	0.596	1.000				0.637	0.821
	RC2	0.587	1.161	0.292	3.972	***	0.751	
	RC3	0.544	1.330	0.353	3.770	***	0.929	
Self-efficacy (SE)	SE1	0.696	1.000				0.749	0.784
	SE2	0.643	0.644	0.199	3.235	**	0.621	
	SE3	0.705	1.272	0.364	3.493	***	0.840	
Participation behavior intention (PBI)	PBI1	0.811	1.000				0.803	0.853
	PBI2	0.670	0.795	0.066	12.007	***	0.807	
	PBI3	0.739	0.875	0.074	11.896	***	0.825	
participation behavior (PB)	PB1	0.790	1.000				0.810	0.876
	PB2	0.683	0.822	0.101	8.127	***	0.865	
	PB3	0.881	1.148	0.122	9.387	***	0.837	

S.E. is Standard Error, C.R. is Critical Ratio, ρ_c_ is Composite Reliability, Significant at *** P ≤ 0.01, **P ≤ 0.05.

**TABLE 5:** Villager participation model differential validity test.

Latent variable	AVE	Cronbach’s α	PB	PBI	SE	RC	PA	SN	PN	SEP	NEP
PB	0.702	0.926	0.838								
PBI	0.659	0.954	0.800	0.812							
SE	0.551	0.773	0.471	0.306	0.742						
RC	0.611	0.808	0.486	0.662	0.319	0.782					
PA	0.464	0.716	0.622	0.635	0.214	0.664	0.681				
SN	0.626	0.814	0.631	0.454	0.303	0.380	0.655	0.791			
PN	0.760	0.901	0.265	0.376	0.190	0.726	0.598	0.404	0.872		
SEP	0.596	0.843	0.255	0.488	0.484	0.565	0.496	0.395	0.604	0.772	
NEP	0.505	0.764	0.368	0.601	0.400	0.461	0.660	0.554	0.434	0.626	0.711

*The numbers on the diagonal of the table are the square root of the AVE value.*

### Goodness-of-Fit Test for Measurement Model and Structure Relation Model

In terms of the goodness-of-fit test of the villager participation behavior measurement model, the AVE quantity of the PA1 standardized coefficient participation attitude fell short of the requirement. In other words, although some indicators in the PA measurement model did not satisfy the requirement, they were a close fit. Therefore, the overall degree of fitting of the measurement model was consistent with the requirements, as shown in Table 6. The chi-square value (CMIN) of the model was 599.83, the degree of freedom (DF) of the model was 295, and its chi-square degree of freedom ratio (CMIN/DF) was 2.033, indicating that the model had a good degree of fit. The SRMR was 0.051. Although it did not meet the standard of <0.05, the difference in value was very small. Thus, it was considered to meet the standard level. The root mean squared error of approximation met the requirement of being less than 0.08. The parsimony normed fit index (NFI), Trucker-Lewis index (TLI), the comparative fit index (CFI), and the incremental fit index (IFI) all satisfy the criterion greater than 0.9. Therefore, the model has a good fit.

**TABLE 6:** Adaptation analysis of villagers’ participation in micro-landscape construction model.

Fitting indicator	CMIN/DF	SRMR	RMSEA	NFI	TLI	CFI	IFI
Measurement standard	1< CMIN/DF >3	≤0.05	<0.08	>0.9	>0.9	>0.9	>0.9
Model indicator value	2.033	0.051	0.05	0.93	0.914	0.947	0.942

### Hypothesis Test

**FIGURE 2:**
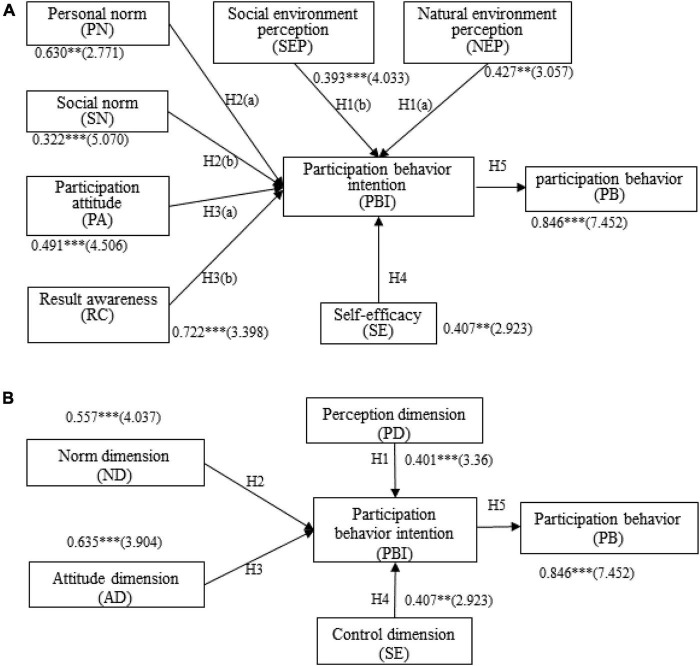
Villagers participate in the test results of rural micro-landscape construction behavior model **(A,B)**. * indicates a significant level *P* < 0.05, ^**^ indicates a significant level *P* < 0.01, ^***^ indicates a significant level *P* < 0.001; the number in parentheses is the corresponding *t* value.

By combining the measurement model with the structure relation model test, the standardized structure model verified that the standardized load of the observed variables was in the range of 0.322–0.722, which was significant at the level of 0.01. As shown in Figures 2A,B, H1, H1(b), H2, H2(b), H3, H3(a), H3(b), and H5 were significant at a level of 0.001, while H1(a), H2(a), and H4 were significant at a level of 0.01. To determine whether different independent variables played a role in mediating variables, we adopted the bootstrap method to investigate the indirect effects of different independent variables on PBI. According to the results, the 95% confidence interval of the indirect effect estimate value among all independent variables (e.g., NEP, SEP, PN, SN, PA, result awareness, and SE), as well as PBI and participation behavior, excluded 0, indicating that the mediating effect was significant (Zhao et al., 2010), as shown in Table 7. This confirmed the ninth hypothesis of this study.

**TABLE 7:** Results of hypothesis testing for the structural model.

	Influence path			Unstandardized estimate	S.E.	C.R.	*P*	Standardized estimate	Status
H1	PBI	←	PD	1.804	0.537	3.36	***	0.401	Supported
H1(a)	PBI	←	NEP	1.920	0.628	3.057	**	0.427	Supported
H1(b)	PBI	←	SEP	1.908	0.473	4.033	***	0.393	Supported
H2	PBI	←	ND	1.780	0.441	4.037	***	0.557	Supported
H2(a)	PBI	←	PN	3.541	1.278	2.771	**	0.630	Supported
H2(b)	PBI	←	SN	2.180	0.430	5.070	***	0.322	Supported
H3	PBI	←	AD	1.714	0.439	3.904	***	0.635	Supported
H3(a)	PBI	←	PA	1.334	0.296	4.506	***	0.491	Supported
H3(b)	PBI	←	RC	2.902	0.854	3.398	***	0.722	Supported
H4	PBI	←	SE	1.090	0.373	2.923	**	0.407	Supported
H5	PB	←	PBI	0.827	0.111	7.452	***	0.846	Supported

* indicates a significant level P < 0.05, ** indicates a significant level P < 0.01 and *** indicates a significant level P < 0.001. The number in parentheses is the corresponding t value. PBI is participation behavior intention, NEP is natural environment, SEP is social environment, PN is personal norm, SN is social norm, RC is result awareness, PA is participation attitude, SE is self-efficacy, PD is perception, ND is norm dimension, AD is attitude dimension.

## Results and Analysis

### Perception Dimension

Perception refers to a series of actions carried out with an awareness of internal and external environments. In the PD, the results of the model analysis confirmed H1(a) and H1(b). Specifically, the perception of the natural environment positively affected the willingness to participate in behavior at a significance level of 0.001, and a willingness to participate in behavior had an indirect effect on participation. That is, for each additional unit of NEP, the willingness to participate in a particular behavior increased by 0.427 units. At the 0.001 significance level, SEP positively affected the willingness to participate in a particular behavior and had an indirect effect on participation behavior through the willingness to participate in the behavior. In the PD, SEP had a greater impact on willingness to participate in behavior than NEP. In terms of the mediation effect, the mediating effect of NEP (0.361) was stronger than that of SEP (0.332). The villagers’ perception of their living environment reflected their realistic needs for ecological livability. On the one hand, from the perspective of urban–rural integration, there is room for improvement in the rural environment and landscape, with villagers being particularly worried about their living environment. On the other hand, a good social atmosphere can effectively promote communication among villagers, which is conducive to the establishment of civil environmental landscape organizations, thereby enhancing the enthusiasm of villagers to participate in the construction of rural landscapes.

### Norm Dimension

Norm refers to an individual’s code of conduct toward oneself or others (Terry et al., 1999). In the norm dimension, from the results of the model analysis, H2(a) and H2(b) were confirmed. Specifically, PN produced a positive influence on PBI at a significance level of 0.01 and exerted indirect effects on participation behavior by virtue of PBI. SN perception had a positive effect on PBI at a significance level of 0.001 and exerted indirect effects on participation behavior by virtue of PBI. In the norm dimension, the influence of PN (0.630) on PBI was greater than that of the SN (0.322). In terms of the mediation effect, the mediating role of personal norms (0.53) was greater than that of social norms (0.272). This may be because both personal and social norms are “soft constraint” procedures, making villagers to not only exercise self-discipline but also supervise each other. Once an individual violates rural value recognition and common norm behaviors, they inevitably become subject to rejection and moral condemnation from other members.

### Attitude Dimension

In the attitude dimension, the model analysis results confirmed H3(a) and H3(b). Specifically, PA positively influenced PBI at a significance level of 0.001, exerting indirect effects on participation behavior by virtue of PBI. Result awareness positively affected PBI at a significance level of 0.001, exerting indirect effects on participation behavior by virtue of PBI. The influence of result awareness (0.722) on PBI was greater than that of PA (0.491). With regards to the mediation effect, the mediating role of result awareness (0.611) was greater than that of PA (0.415). With the transformation of rural society from traditional to modern, the improvement in villagers’ subjectivity will continuously strengthen their sense of ownership of rural governance. When villagers assume the governance of the rural environment as their responsibility and obligation, their intention to participate in the construction of the rural micro-landscape will be reinforced accordingly.

### Control Dimension

In terms of the control dimension, H4 was confirmed. Specifically, SE was found to have a positive effect on PBI at a significance level of 0.01, exerting indirect effects on participation behavior based on PBI. In terms of the mediation effect, the mediating effect of SE on participation behavior was 0.344. This indicates that, with the improvement of villagers’ awareness of rural landscape construction, villagers become increasingly convinced of the important impact of the rural landscape environment on their lives and are willing to make an effort to create a healthy rural living environment. In addition, this positive self-control ability naturally encourages villagers to participate in the construction of rural micro-landscapes.

As shown in Table 8, the effect intensity of the four dimensions on villager PBI in the new first-order structure equation model was as follows, from highest to lowest: attitude dimension (0.635) > norm dimension (0.557) > control dimension (0.407) > PD (0.401).

**TABLE 8:** Mediation bootstrapping test.

Indirect effects	Estimate	95%CI		Conclusion
		
		Lower	Upper	
NEP→PBI→ PB	0.173	1.621	2.768	Mediation
SEP→ PBI → PB	0.196	0.123	3.931	Mediation
PN→PBI → PB	0.716	0.362	1.293	Mediation
SN→PBI → PB	0.125	1.023	1.376	Mediation
PA→PBI → PB	0.543	0.652	2.513	Mediation
RC→PBI → PB	0.989	0.183	2.496	Mediation
SE→PBI → PB	0.107	1.068	1.66	Mediation

## Conclusion

From the perspective of personal behavior, this study combines TPB and the norm activation theory to construct an influencing factor model of villagers’ participation behavior in rural micro-landscape construction from four dimensions: perception, norm, attitude, and control. An empirical analysis was conducted using survey data of 414 villagers in Jinjiang, and the following conclusions were drawn: (1) the model integrating the norm activation theory and the behavior theory can better account for the influencing mechanism of villager participation in the rural micro-landscape construction model; (2) through the specific analysis of the influencing factors of villager participation in rural micro-landscape construction, the perception, norm, attitude, and control dimensions were all found to have a significant effect on participation behavioral intention. Among them, the order of the degree of influence was as follows: attitude, norm, control, and perception dimensions. In the attitude dimension, the influence of result awareness on participation behavior was greater than that of PA. In the norm dimension, the influence of personal norms on participation behavior was greater than that of social norms. In the PD, the influence of NEP on participation behavior was greater than that of SEP; and (3) based on the mediation results, NEP, SEP, PN, SN, PA, result awareness, and SE all exerted indirect effects on participation behavior through the villagers’ PBI. The order of the strength of the mediating effect was as follows: result awareness, PN, PA, natural environment awareness, SE, social environment awareness, and SN.

### Policy Suggestions

Based on the above conclusions, we propose the following policies to promote villager participation in the construction of rural micro-landscapes:

(1) The construction of a bidirectional incentive mechanism based on material and personal incentives to promote villager participation in the norm dimension of rural micro-landscape construction. Specifically, positive incentive measures are advocated, such as monetary rewards and enhanced social status. Regarding destructive behaviors against micro-landscapes, measures such as criticism and fines can be adopted to establish effective negative incentives.
(2) The establishment of a rural information disclosure system to reinforce villagers’ participation awareness and attitudes toward rural micro-landscape construction. The government should rigorously enforce laws to guarantee the rights of villagers to participate in rural micro-landscape construction. Additionally, the government will need to broaden the means of villager participation to ensure that villagers can participate in the construction of rural micro-landscapes extensively and efficiently.
(3) The stimulation of villagers’ SE through targeted guidance, including strengthening the education and training of villagers, promoting their self-cognition, participation awareness, and participation ability, and encouraging villagers to participate in the construction of rural micro-landscapes.

### Limitations and Future Research

Although villagers’ participation in landscape planning is an emerging field of research, there is an extensive history of achievements in research on public participation. From the perspective of sustainable development goals, it is clear that rural micro-landscapes with villager participation better promote the sustainable development of rural areas, accelerate the construction of attractive villages, and achieve the overall goal of rural revitalization. However, research on villager participation in rural landscape construction is a comprehensive process. As far as this field of research is concerned, due to the limitations of time and labor, this study has several limitations. The construction of rural micro-landscapes involves complex system engineering that integrates multiple fields and disciplines. This is further complicated by the fact that different regions have different economic cultures. Although the current micro-landscape construction model in Jinjiang has achieved good results, whether this can be popularized and promoted across China more generally remains to be seen. Therefore, future research will need to be conducted in different study areas, and the resulting similarities and differences in the results of villagers’ participation behavior will need to be analyzed.

## Data Availability Statement

The original contributions presented in this study are included in the article/supplementary material, further inquiries can be directed to the corresponding author.

## Ethics Statement

Ethical review and approval was not required for the study of human participants in accordance with the Local Legislation and Institutional Requirements. Written informed consent from the patients/participants was not required to participate in this study in accordance with the National Legislation and the Institutional Requirements.

## Author Contributions

HC, QR, KY, XL, and JL planned and designed the study. HC, QR, KY, and HC carried out the data collection and processed the data. JL and XL performed the statistical analyses for all outcomes with the advice and assistance. HC wrote both first and consecutive drafts of the manuscript. All authors participated in data interpretation and provided input into the development of the manuscript, read, and agreed to the published version of the manuscript.

## Conflict of Interest

QR was employed by Haixia (Fujian) Transportation Engineering Design Co., Ltd. The remaining authors declare that the research was conducted in the absence of any commercial or financial relationships that could be construed as a potential conflict of interest.

## Publisher’s Note

All claims expressed in this article are solely those of the authors and do not necessarily represent those of their affiliated organizations, or those of the publisher, the editors and the reviewers. Any product that may be evaluated in this article, or claim that may be made by its manufacturer, is not guaranteed or endorsed by the publisher.
